# Effects of the Application of Virtual Reality to Experiential Education on Self-Efficacy and Learning Motivation of Social Workers

**DOI:** 10.3389/fpsyg.2021.770481

**Published:** 2021-10-27

**Authors:** Suh Chen Hsiao

**Affiliations:** Department of Adult Mental Health and Wellness, USC Suzanne Dworak-Peck School of Social Work, University of Southern California, Los Angeles, CA, United States

**Keywords:** virtual reality, experiential education, social worker, self-efficacy, learning motivation

## Abstract

To enhance the human resources required for national development to cope with the change, countries in the world have positively invested in education, as national education in the future is necessary to cultivate new-generation citizens with new traits and abilities to cope with the possible impacts and challenges in the new century. For this reason, the education reform wave sweeps many countries. The experiential learning model in experiential education nowadays leads profit and non-profit organizations in the business community, education, and social worker groups to the alternative education trend. Various experiential learning curricula are therefore spread. Taking social workers in southern Taiwan as the research objects, a total of 227 social workers are preceded the 15-week (3 h per week for a total of 45 h) experimental research with the application of virtual reality to experiential education. The research results summarize that (1) experiential education with virtual reality would affect self-efficacy, (2) experiential education with virtual reality would affect learning motivation, and (3) self-efficacy reveals remarkably positive effects on learning motivation. According to the results, it is expected to increase the interaction among the social workers through the learning activity and internalize the experience in the practical learning process of communication, problem solving, and extrinsic interaction for the application to the work to achieve a better life.

## Introduction

In the 21st century, people are in the era with rich information, advanced technology, rapidly changing society, convenient life, and close international relationship, but fierce international competition. To enhance the human resources required for national development to cope with such changes, countries in the world have positively made a huge investment in education as it is necessary for future national education to cultivate new-generation citizens with the new traits and abilities to cope with the possible impacts and challenges in the new century. As a result, the education reform wave sweeps many countries. Experiential education activity combines the principles and methods of natural education, field trip, and experiential courses and is promoted to schools, social education, medical care, leisure, and education guidance in legal affairs units (Chapman et al., 1992). The essence of education could be understood through the development background and process. Experiential education provides purposive active learning opportunities through real situations to strengthen the individual growth and the interactive operation and response-ability of organizations through individual and group interactive learning (Kwon, 2019). Each person plays a different role, as important as a screw of a machine, in the experiential education process. Team training could rapidly cultivate the team spirit of participants and constantly produce peak experience of participants in learning so that learners could fast grasp the learning objectives and members could receive different learning from the past in the future and life. The experiential learning model in experiential education nowadays could lead profit and non-profit organizations in the business community, education, and social worker groups to the trend of alternative education. In this case, various experiential learning curricula are broadly spread. Leonard (1990) stated that the experiential education activity is explained with different terms, such as exploratory education and adventure education. Experiential education is getting started domestically in the past few years. The above-mentioned experiential education covers excitement, uncertainty, reality, perceived risk, effort, and mutual effect with the natural environment so that the enterprises change or adjust the past taught courses in the personnel training into experiential education curricula to increase the fun. In this case, the effects of the application of virtual reality to experiential education on the self-efficacy and learning motivation of social workers are discussed in this study, expecting to increase the interaction among the social workers through such learning activity and internalize the experience in the practical learning process of communication, problem solving, and extrinsic interaction to the work to achieve the better life.

## Literature Review and Hypothesis

### Literature

Huang and Liaw (2018) mentioned the value of experiential education, such as learning by doing, reflection, teamwork, autonomous challenge, problem solving, the establishment of trust, and listening and expression, and discovered that after integrating experiential education into curricula, the problem-solving ability of students, team cohesiveness, extrinsic interaction, frustration tolerance, character education, and self-concept would be improved. Breunig (2019) explained experiential education as the learning method integrating explored events, issues, or tasks into the activities to guide the experience, perception, insight, comprehension, and application of the participants. Experiential education created learning situations and provided self-experience and team experience opportunities for people to find out personal ability, value, enthusiasm, and responsibility. Ye et al. (2019) indicated that stressing the learning process of students in experiential education, maintaining a good teacher-student relationship with interaction and positive feedback, and carefully observing the responses of students with proper guidance to cultivate active problem thinking behavior of students could improve the self-efficacy of the students and team cohesiveness after the end of the courses. The following hypothesis is therefore proposed in this study.

Chang et al. (2018) mentioned the significant effect of participation in experiential education on the enhancement of self-concept and self-fulfillment to promote the self-concept and self-fulfillment with continuous learning motivation. Falloon (2019) considered that experiential education could enhance the learning motivation and learning effectiveness of general students. Zheng et al. (2018) regarded the objective of experiential education as allowing students to practice, experiencing, and reflecting the meaning of life through the curriculum. The activity curriculum was the integrated curriculum based on the learning motivation of the students; in experiential education, the activities were more than lectures, with certain flexibility in the materials and process, emphasizing the individual differences, problem solving centered, and aiming to cultivate the problem-solving ability of the students. Accordingly, the following hypothesis is proposed in this study.

Cheng and Tsai (2019) pointed out the function of self-efficacy in the individual decision of learning motivation to appear significant effects on the learning process and effect. People with higher self-efficacy presented more frequent self-judgment behavior and better self-learning motivation. Chang et al. (2019) revealed that pupils with stronger self-efficacy would enhance the individual self-confidence through the feedback of learning experience, self-evaluation process, and learning outcome on self-efficacy to present higher learning motivation. Hwang et al. (2019) mentioned that some studies pointed out the remarkably positive correlations between self-efficacy and learning motivation that the higher self-efficacy, the higher learning motivation. The following hypothesis is further proposed in this study.

### Research Hypothesis

According to the above literature, the following hypotheses are proposed in this study.

H1: Experiential education with virtual reality would affect self-efficacy.
H2: Experiential education with virtual reality would affect the motivation to learn.
H3: Self-efficacy shows notably positive effects on the motivation to learn.

## Methodology

### Measurement of Research Variable

#### Experiential Education With Virtual Reality

Li (2014) mentioned that experiential education should present several important dimensions of “challenge,” “team,” and “self-reflection.” They are applied to this study.

1. Challenge: Experiential education activity should present challenges and fun to attract the participation of members.
2. Team: Experiential education is preceded by teams, and the members in the activity must be participants who could engage in the activity according to their ability and needs.
3. Self-reflection: After experiencing a series of designed activities, the members must precede reflection, digest, and absorb the physical, psychological, and spiritual perception and experience, recombine, and internalize into meaningful gains for themselves, and change the behavior.

#### Self-Efficacy

Referring to Lei et al. (2019), the self-efficacy in this study contains three dimensions.

1. Cognitive influence: People with higher self-efficacy present higher ambition and longer points of view are more thoughtful, and more willing to accept the difficult challenges and would firmly devote themselves to those challenges.
2. Motivational influence: The belief in self-efficacy to be able to complete certain affairs would affect people's goal setting, action strategy, willingness to make efforts, persistence to face a challenge, and degree of recovery from frustration.
3. Affective influence: The bearable pressure, when people encounter dilemmas or threats, is mostly decided by the degree of their consideration of completing the affair.

#### Learning Motivation

According to the research of Chen et al. (2019), learning motivation is divided into two dimensions in this study.

1. Intrinsic orientation: Including favor of challenging courses, regarding learning as interest and hobby, considering that learning could expand vision, actively learning new courses, and learning for developing self-potential and fulfilling ideal.
2. Extrinsic orientation: Covering learning for receiving others' affirmation, obtaining better performance, passing examinations or evaluation, showing off to others, competing with classmates, receiving appreciation and attention from elders or the opposite sex, avoiding punishment and scold, and preventing from the shame of failure.

### Research Subject

Taking social workers in southern Taiwan as the empirical objects, a total of 227 social workers are preceded the experimental research on experiential education with virtual reality. After deducting invalid and incomplete copies of the questionnaire, 216 valid copies are retrieved, with a retrieval rate of 95%.

### Analysis Method

Both SPSS 22.0 and AMOS 20.0 (IBM Corp., NY, USA) are used for the analysis. The structural model in the structural equation model (SEM) is applied to confirm the effects of experiential education with virtual reality, self-efficacy, and learning motivation and test the hypotheses.

### Experimental Design and Process

This study aimed to discuss the effect of the virtual reality applied experiential education on the self-efficacy and motivation to learn of the social workers, with experimental design. The experiential education with virtual reality is preceded with experiments, and the self-efficacy scale and motivation to learn scale are used as the testing tools for the 24-week (2 h per week for a total of 48 h) experimental research.

Groups, such as the experimental group and control group, are the independent variables, and self-efficacy and motivation to learn are the dependent variables in this study.

#### Independent Variable

The independent variables, the experimental group, are preceded the experiment. The experimental group applies “experiential education with virtual reality.”

#### Dependent Variable

Dependent variables in this study refer to the post-test performance of the subjects on the “self-efficacy scale” and “motivation to learn scale.”

## Result of the Study

### Factor Analysis

The experiential education with virtual reality scale, after factor analysis, extracted three factors of “challenge” (eigenvalue = 2.841, α = 0.86), “team” (eigenvalue = 1.975, α = 0.82), and “self-reflection” (eigenvalue = 1.637, α = 0.85). The cumulative covariance explained achieves 72.438%. The self-efficacy scale, after factor analysis, extracted three factors of “cognitive influence” (eigenvalue = 2.664, α = 0.87), “motivational influence” (eigenvalue = 2.136, α = 0.88), and “affective influence” (eigenvalue = 1.845, α = 0.90). The cumulative covariance explained reaches 77.253%. The learning motivation scale, after factor analysis, is extracted two factors of “intrinsic orientation” (eigenvalue=3.514, α =0.91) and “extrinsic orientation” (eigenvalue=3.193, α =0.92). The cumulative covariance explained achieves 81.624%.

### Correlation Analysis

From Table 1, experiential education with virtual reality, self-efficacy, and learning motivation show significant correlations, revealing that H1, H2, and H3 are preliminarily supported.

**Table 1:** Correlation analysis.

**Research dimension**	**α**	**Experiential education with virtual reality**	**Self-efficacy**	**Learning motivation**
Experiential education with virtual reality	0.84			
Self-efficacy	0.89	0.33^**^		
Learning motivation	0.91	0.38^**^	0.31^**^	

*Note*:

** *p < 0.01*.

### Evaluation Indicators of SEM

The model fit could be evaluated from the preliminary fit criteria, overall model fit, and fit of the internal structure of the model. The research data are organized as below.

From the complete model analysis result, three dimensions of experiential education with virtual reality (challenge, team, and self-reflection) could remarkably explain experiential education with virtual reality (*t* > 1.96, *p* < 0.05), three dimensions of self-efficacy (cognitive influence, motivational influence, and affective influence) could notably explain the self-efficacy (*t* > 1.96, *p* < 0.05), and two dimensions of learning motivation could significantly explain the learning motivation (*t* > 1.96, *p* < 0.05). Apparently, the overall model in this study presents good preliminary fit criteria.

In terms of internal fit, experiential education with virtual reality reveals positive and remarkable correlations with self-efficacy (0.392, *p* < 0.01), self-efficacy appears positive and notable correlations with learning motivation (0.367, *p* < 0.01), and experiential education with virtual reality shows positive and significant correlations with learning motivation (0.433, *p* < 0.01) that H1, H2, and H3 are supported.

Regarding overall model fit, the overall model fit standards χ^2^/df = 1.721, smaller than the standard 3, and root mean squared residual (RMR) = 0.004, revealing the proper results of χ^2^/df and RMR. Furthermore, chi-square is sensitive to the sample size that it is not suitable for directly judging the fit. However, the overall model fit standards goodness of fit index (GFI) = 0.968 and adjusted goodness of fit index (AGFI) = 0.917, higher than the standard 0.9 (the closer GFI and AGFI to 1 revealing the better model fit) that this model presents better fit indices. The hypothesis test results are shown in Table 2.

**Table 2:** Hypothesis test.

**Research hypothesis**	**Correlation**	**Empirical result**	**P**	**Result**
H1	+	0.392	0.00	Supported
H2	+	0.367	0.00	Supported
H3	+	0.433	0.00	Supported

**Figure 1:**
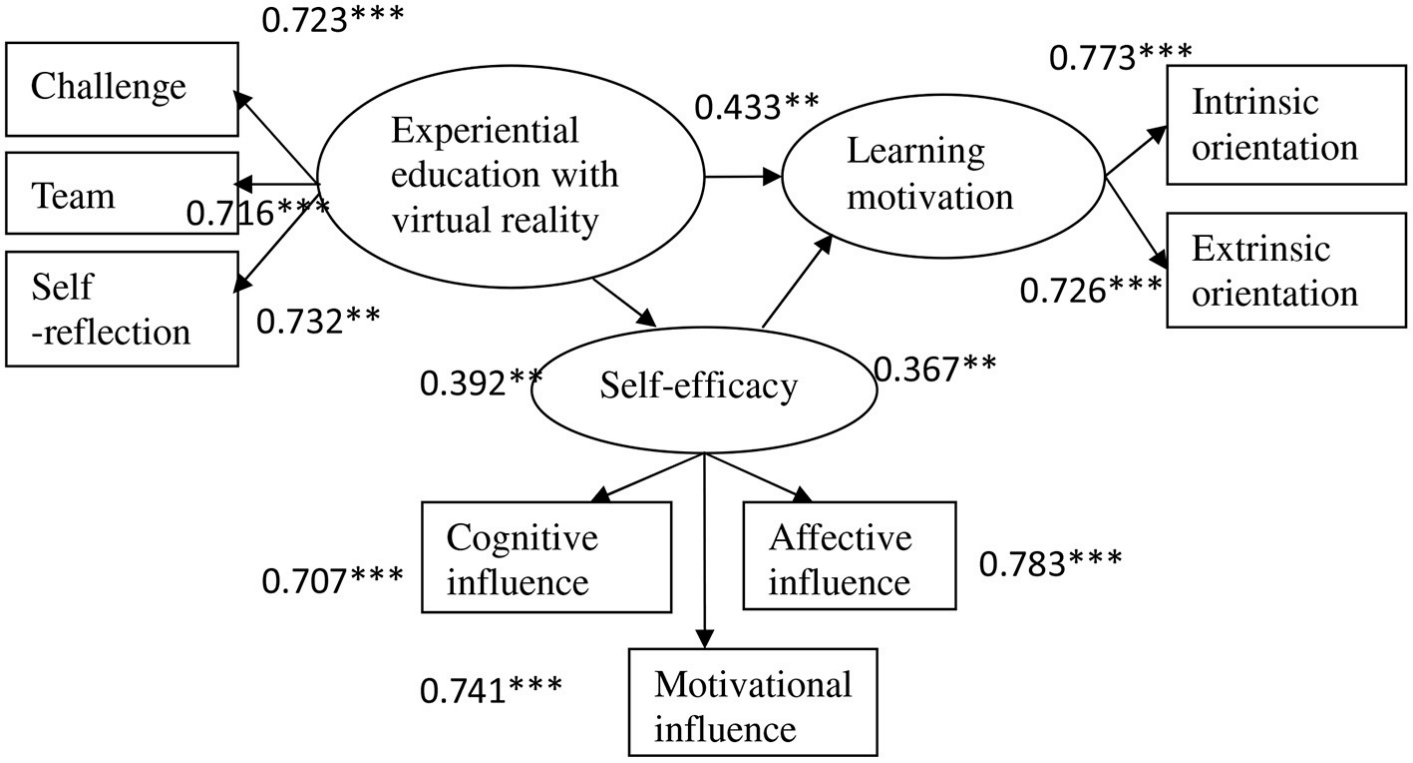
The path coefficients analysis. **p < 0.01 and ***p < 0.001.

Figure 1 shows the overall research result. The path coefficients achieving the significance are denoted with solid lines, while those without reaching the significance are shown with dotted lines. The path coefficients of variables achieve significance, revealing the convergent validity of such path coefficients. It is the basic requirement for model analysis. Accordingly, the model fit is verified, i.e., the research model conforming to the theory with validity.

## Discussion

The idea of applying virtual reality to an experiential learning model is to organize and induce specific and meaningful environmental experiences or events through the inner reflection and thinking of social workers to eventually generate an abstract idea or model. When there is a similar experience in future events and environment, the social workers, with an individual will, could select to apply the ideas from past experiences and re-establish and learn new concepts. The research results are similar to the research results of Glass and Benshoff (2002), and Sibthorp and Arthur-Banning (2004). Motivation to learn is the inner mental process of learners appearing drive on the participated learning activities and continuously preceding the learning activities and allows learners to approach the goals set by instructors (Brown, 2004). The stronger motivation to learn would naturally result in better learning effectiveness. When someone tries to learn, environment, behavior, and the mutual interaction between individuals will all take part in affecting the outcome (Paisley et al., 2008; Thomas, 2008) and, aiming at specific tasks or curricula, the subjective evaluation of the ability to complete tasks (Paisley et al., 2008). For this reason, the self-efficacy of the learners would affect the problem-solving motivation and ability in the learning activities; different curriculum designs would be planned according to the self-efficacy of the learners. Nevertheless, even experienced teachers might appear blind spots in the actual teaching process. The application of virtual reality to experiential learning could help teachers understand the learning state of the social workers from the feedback and the opinions and learning experience of the participatory social workers on the worksheets. Besides, the guidance and teaching effect could be strengthened aiming at an individual and the other special situations. It could achieve the effect of two-way communication and assist teachers in adjusting and correcting the curricula or teaching styles.

The teachers with rich experiences might appear blind spots in the actual teaching process.

## Conclusion

An experiential learning model introducing experience into personal learning is now broadly applied to schools, society, enterprises, and psychological counseling. With the factor of self-challenge, it could be applied to youth recovery, the life effectiveness of college athletes, problem-solving ability, knowledge promotion of the teachers, learning abilities of pupils, corporate team development, and physical image to achieve the estimated effectiveness. The application of virtual reality to experiential education is therefore selected for this study. The research results show that the social workers with higher self-efficacy in the application of virtual reality to experiential education would enhance the learning motivation. The research results are similar to those of McKenzie (2003); Goldenberg and Pronsolino (2008), and Sibthorp et al. (2011). Possibly because the application of virtual reality to experiential education is more flexible and the operation process is relaxing that the social workers could learn in a relaxed mood. In this case, higher self-efficacy would enhance the learning motivation.

## Data Availability Statement

The original contributions presented in the study are included in the article/supplementary material, further inquiries can be directed to the corresponding author/s.

## Ethics Statement

The present study was conducted in accordance with the recommendations of the Ethics Committee of the University of Southern California, with written informed consent being obtained from the participant. The participant was asked to read and approve the ethical consent form before participating in the present study. The participant was also asked to follow the guidelines in the form in the research. The research protocol was approved by the Ethical Committee of the University of Southern California, CA, USA.

## Author Contributions

SH revised and approved the submitted version of the manuscript.

## Conflict of Interest

The author declares that the research was conducted in the absence of any commercial or financial relationships that could be construed as a potential conflict of interest.

## Publisher's Note

All claims expressed in this article are solely those of the authors and do not necessarily represent those of their affiliated organizations, or those of the publisher, the editors and the reviewers. Any product that may be evaluated in this article, or claim that may be made by its manufacturer, is not guaranteed or endorsed by the publisher.
